# Total Flavonoids from *Camellia oleifera* Alleviated *Mycoplasma pneumoniae*-Induced Lung Injury via Inhibition of the TLR2-Mediated NF-κB and MAPK Pathways

**DOI:** 10.3390/molecules28207077

**Published:** 2023-10-13

**Authors:** Nan Ding, Aihua Lei, Zhisheng Shi, Lin Xiang, Bo Wei, Yimou Wu

**Affiliations:** 1 Hunan Provincial Key Laboratory for Special Pathogens Prevention and Control, Hunan Province Cooperative Innovation Center for Molecular Target New Drug Study, Institute of Pathogenic Biology, Hengyang Medical College, University of South China, Hengyang 421001, Chinaleiaihua18@usc.edu.cn (A.L.);; 2 Research Lab of Translational Medicine, Hengyang Medical College, University of South China, Hengyang 421001, China

**Keywords:** flavonoids, *Mycoplasma pneumoniae*, *Camellia oleifera*, TLR2, NF-κB, MAPK

## Abstract

*Mycoplasma pneumoniae* (*M. pneumoniae*) is an atypical bacterial pathogen responsible for community-acquired pneumonia primarily among school-aged children and young adults. *Camellia oleifera* (*C. oleifera*) has been used as a medicinal and edible plant in China for centuries, the constituents from which possessed various bioactivities. Notably, flavonoids existing in residues of *C. oleifera* defatted seeds exhibited significant anti-inflammatory activities. In the present study, we investigated the impact of total flavonoids from *C. oleifera* (TFCO) seed extract on *M. pneumoniae* pneumonia. TFCO was obtained using multiple column chromatography methods and identified as kaempferol glycosides via UPLC-HRESIMS. In a *M. pneumoniae* pneumonia mouse model, TFCO significantly reduced the lung damage, suppressed IL-1β, IL-6, and TNF-α production, and curbed TLR2 activation triggered by *M. pneumoniae*. Similarly, in RAW264.7 macrophage cells stimulated by lipid-associated membrane proteins (LAMPs), TFCO suppressed the generation of proinflammatory cytokines and TLR2 expression. Moreover, TFCO diminished the phosphorylation of IκBα, JNK, ERK, p38, and p65 nuclear translocation in vitro. In conclusion, TFCO alleviated *M. pneumoniae*-induced lung damage via inhibition of TLR2-mediated NF-κB and MAPK pathways, suggesting its potential therapeutic application in *M. pneumoniae*-triggered lung inflammation.

## 1. Introduction

*Mycoplasma pneumoniae* (*M. pneumoniae*) stands out as an atypical bacterial pathogen, manifesting primarily as the lead agent for community-acquired pneumonia (CAP) among school-aged children and young adults [1]. *M. pneumoniae* prompts inflammation in the upper and lower respiratory tracts and contributes to a broad spectrum of extrapulmonary syndromes [2,3,4,5]. Accumulating evidence has confirmed that *M. pneumoniae*-induced pneumonia is closely associated with an elevated production of proinflammatory cytokines, including tumor necrosis factor-*α* (TNF-*α*) and interleukin- (IL-) 1β and IL-6. These molecules are not merely byproducts but central actors that cause a series of complex immune events. Their mobilization and activation of an array of leukocytes, such as macrophages and neutrophils, can be a double-edged sword—while they mount a defense against invading pathogens, they can also perpetrate tissue damage if not properly regulated [6,7].

Proinflammatory cytokine secretion during *M. pneumoniae* infection correlates with various immune cells, particularly macrophages [8,9,10]. The unusual feature of *M. pneumoniae*—its absence of a cell wall—places the lipid-associated membrane proteins (LAMPs) under the investigative spotlight [7]. These surface proteins, integral to *M. pneumoniae*’s identity, can readily bind to the Toll-like receptor 2 (TLR2) on immune cells, unleashing a cascade of intracellular events [7,11,12]. This involves the activation of signaling pathways like NF-κB and MAPK, which act as conduits to produce a plethora of proinflammatory cytokines [7,13,14]. These pathways, while vital for immune responses, also underscore the delicate balance that needs to be maintained to prevent overactive inflammation [7].

*Camellia oleifera* (*C. oleifera*), a botanical treasure native to regions of central and southern China, has historically been more than just a plant. Its seeds have served in high-quality oil production for centuries. *C. oleifera* by-products, such as camellia seed cake and fruit shell, are extensively used in the dyeing, papermaking, chemical fiber, textile, organic fertilizer, detergent, and pesticide industries [15,16]. Beyond its industrial utility, contemporary research has ventured into tapping its therapeutic potential. The residual defatted seed pomace of *C. oleifera* is rich in bioactive substances, predominantly flavonoids [17,18,19,20,21], saponins [22,23], and polysaccharides [24,25]. Among these, flavonoids, principally kaempferol glycosides, manifest a broad spectrum of biological activities, encompassing anti-inflammation [20,21,26], anti-oxidation [26,27], anti-melanogenesis [27], and anti-microbial [28] activities.

Prior research indicated that *C. oleifera* defatted seed extracts exhibited notable anti-inflammatory activities in vivo, mitigating carbon tetrachloride-induced hepatotoxicity and nonalcoholic fatty liver disease in rats [29,30]. Furthermore, the flavonoids present in the residues of *C. oleifera* defatted seeds inhibited NO and proinflammatory cytokine production in LPS-stimulated RAW264.7 macrophages, chiefly by suppressing the NF-κB signaling pathway [20,21].

Given this context, we first postulated that the total flavonoids from the extract of defatted *C. oleifera* (TFCO) seeds might alleviate *M. pneumoniae*-induced pneumonia. This study not only corroborates our hypothesis but also delves into molecular intricacies to elucidate the inherent mechanisms.

## 2. Results

### 2.1. The Major Components of TFCO

**Figure 1:**
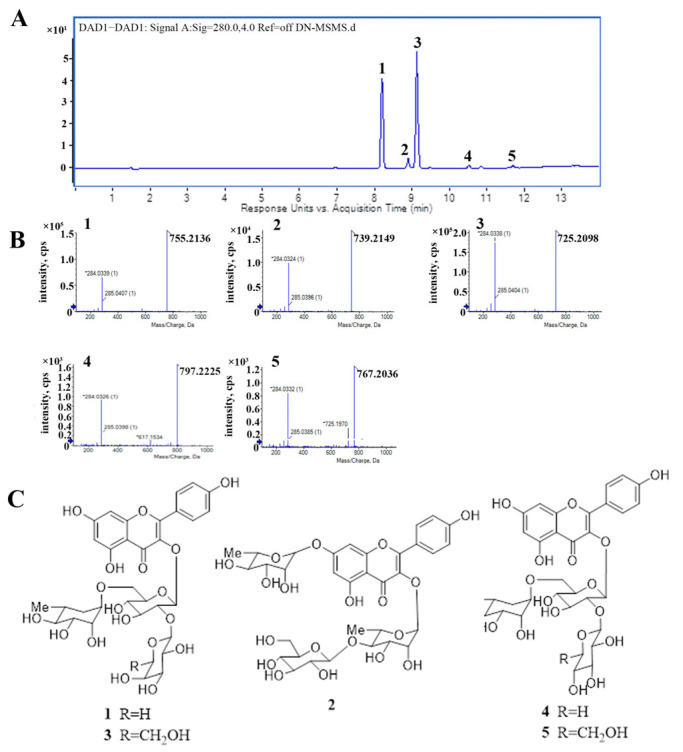
Flavonoids in TFCO. (**A**) UPLC analysis of flavonoids from TFCO; (**B**) HRESIMS spectra of the five kaempferol glycosides from TFCO; (**C**) structures of the five kaempferol glycosides from TFCO.

Five compounds (**1**–**5**) were detected in TFCO by UPLC–HRESIMS and numbered according to the elution order (Figure 1A). The negative HRESIMS for compounds **1**–**5** displayed signals at 755.2136, 739.2149, 725.2098, 797.2225, 767.2036, respectively (Figure 1B). ^1^H NMR (600 MHz) and ^13^C NMR (150 MHz) data for compounds **1** and **3** were consistent with corresponding data reported in the literature (Table S1, Figures S1–S4) [17,18]. The fragment ions at *m/z* 285 indicated the aglycone was kaempferol. These results align with previous reports. Thus, compounds **1**–**5** were confirmed as kaemferol-3-*O*-[2-*O*-β-D-glucopyranosyl-6-*O*-L-rhamnopyranosyl]-β-D-glucopyranoside (compound **1**) [17,18,20], kaempferol-3-*O*-β-D-glucopyranosyl-(1→4)-a-L-rhamnopyranosyl-7-*O*-α-L-rhamnopyranoside (compound **2**) [20,31], kaemferol-3-*O*-[2-*O*-β-D-xylopyranosyl-6-*O*-α-L-rhamnopyranosyl]-β-D-glucopyranoside (compound **3**) [17,18,20], kaempferol-3-*O*-[4″″-*O*-acetyl-α-L-rhamnopyranosyl-(1→6)]-[β-D-glucopyranosyl-(1→2)]-β-D-glucopyranoside (compound **4**) [19], kaempferol-3-*O*-[4″″-*O*-acetyl-α-L-rhamnopyranosyl- (1→6)]-[β-D-xylopyrano-syl-(1→2)]-β-D-glucopyranoside (compound **5**) (Figure 1C) [19]. The UPLC chromatogram revealed that compounds **1**–**5** accounted for 39.44%, 3.78%, 52.95%, 1.31%, and 0.96%, respectively, with a total percentage of 98.44%. The yield and % yield (*w*/*w*) of TFCO and compounds **1**–**5** from *C. oleifera* are listed in Table S2. This suggested that the five kaempferol glycosides were the major constituents present in TFCO.

### 2.2. TFCO Alleviated Pathological Changes in M. pneumoniae-Induced Lung Injury in Mice

**Figure 2:**
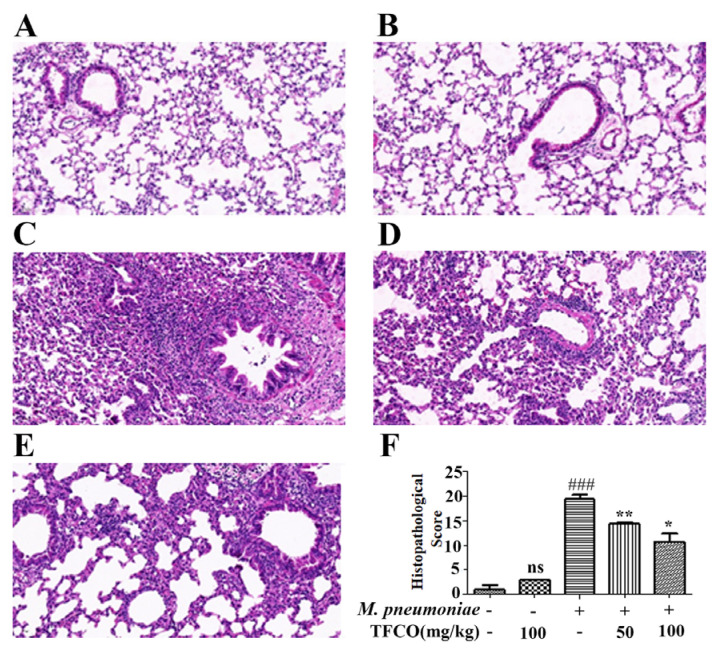
Effects of TFCO on *M. pneumoniae*-induced lung injury. *n* = 5 (**A**) control group; (**B**) TFCO (100 mg/kg) group; (**C**) *M. pneumoniae* group; (**D**,**E**) *M. pneumoniae* + TFCO (50 and 100 mg/kg) groups. (Magnification × 20). (**F**) histopathological scores. ns: non-significant differences, ^###^ *p* < 0.001 versus the control group; * *p* < 0.05, ** *p* < 0.01 versus *M. pneumoniae* group.

The lung sections stained with *Hematoxylin and Eosin* (HE) are shown in Figure 2. No pathological lesions were observed in lung tissues from the control group (Figure 2A) or the TFCO group (Figure 2B). In mice infected with *M. pneumoniae*, lung tissues exhibited significant pathological alterations, including looser alveolar structures, prominent inflammatory cell infiltration, thickened alveolar walls, and narrowed bronchial tubes (Figure 2C). These pathological changes were markedly improved by TFCO administration in a dose-dependent manner (Figure 2D,E). The histology score also indicated that TFCO, isolated from the *C. oleifera* seed extract, could alleviate lung tissue injury caused by *M. pneumoniae* infection (Figure 2F).

### 2.3. TFCO Suppressed Pro-Inflammatory Cytokines Secretion in M. pneumoniae-Infected Mice and LAMPs-Stimulated RAW264.7 Cells without Affecting Cell Viability

**Figure 3:**
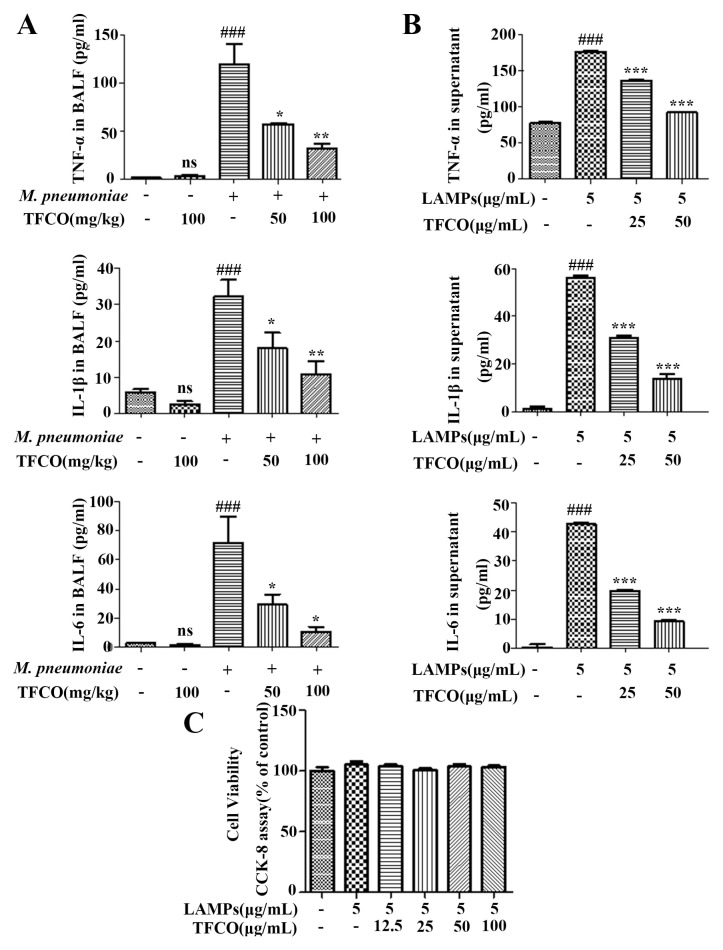
Effects of TFCO on pro-inflammatory cytokines. (**A**) TNF-α, IL-1β, and IL-6 level in BALF of Balb/c mice (*n* = 5). (**B**) TNF-α, IL-1β, and IL-6 level in the supernatants of RAW264.7 cells (*n* = 3). (**C**) cell viability of RAW264.7 cells treated with different concentration of TFCO (*n* = 3). ns: non-significant differences, ^###^ *p*< 0.001 versus control group; * *p* < 0.05, ** *p* < 0.01, *** *p* < 0.001 versus model group.

*M. pneumoniae*-infected mice were analyzed for contents of pro-inflammatory cytokines in the bronchoalveolar lavage fluid (BALF). TNF-α, IL-1β, and IL-6 levels in the *M. pneumoniae* infection group were significantly higher compared to the control group. Treatment with TFCO at 100 mg/kg did not influence TNF-α, IL-1β, and IL-6 levels. In contrast, the content of these pro-inflammatory mediators induced by *M. pneumoniae* infection was markedly decreased by treatment with TFCO at 50 and 100 mg/kg in a dose-dependent manner (Figure 3A). Additionally, ELISA assays demonstrated that TFCO could dose-dependently alleviate the increased TNF-α, IL-1β, and IL-6 levels in the supernatants of LAMPs-stimulated RAW264.7 cells (Figure 3B), while working concentrations of TFCO at 25 and 50 µg/mL had no influence on the cell viability of RAW264.7 cells (Figure 3C).

### 2.4. TFCO Suppressed TLR2 Expression in M. pneumoniae-Infected Mice and LAMPs-Stimulated RAW264.7 Cells

**Figure 4:**
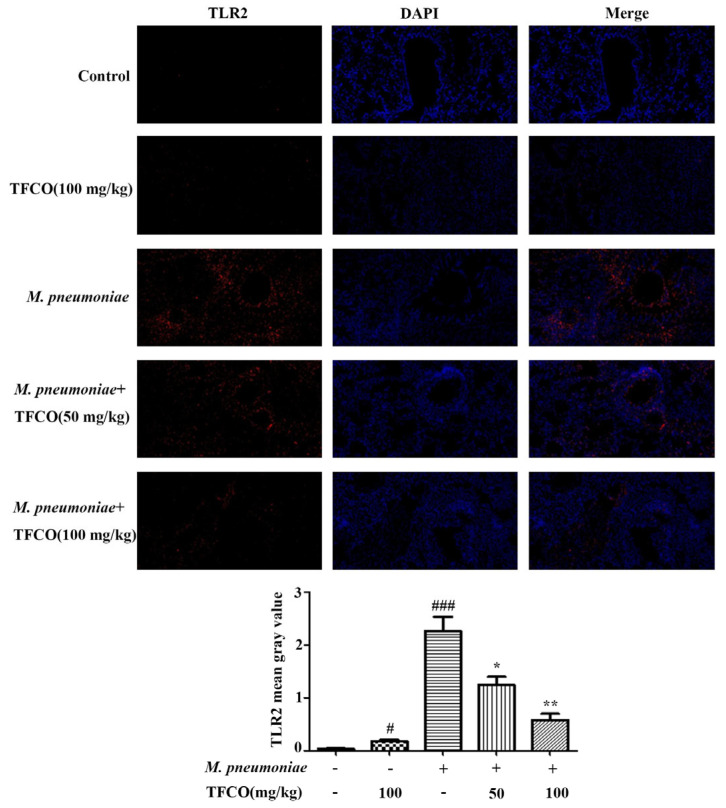
Effects of TFCO on TLR2 in the lung of *M. pneumoniae*-infected Balb/c mice. *n* = 5. Immunofluorescence: DAPI stained blue dots represent nuclei, and the red dots represent TLR2 (Magnification × 20). ^#^ *p* < 0.05, ^###^ *p* < 0.001 versus ctrl group; * *p* < 0.05, ** *p* < 0.01 versus *M. pneumoniae* group.

**Figure 5:**
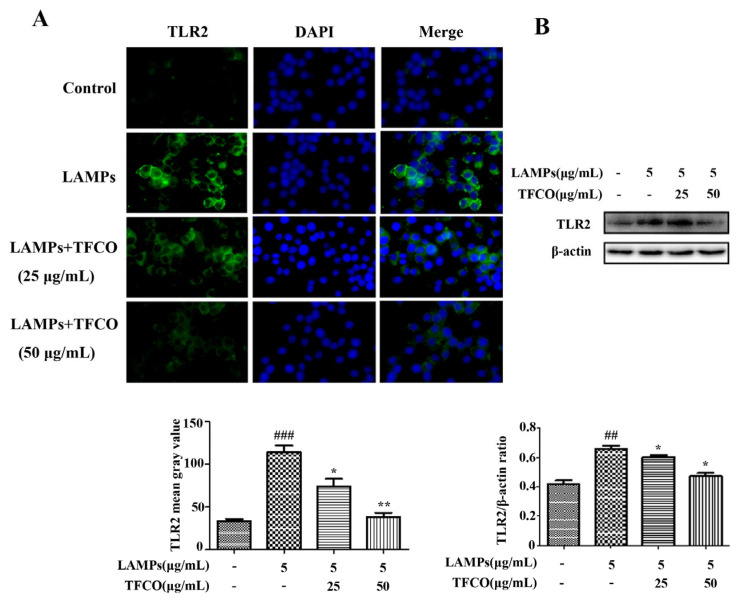
Effects of TFCO on TLR2 in LAMPs-stimulated RAW264.7 cells. (**A**) immunofluorescence: DAPI stained blue dots represent nuclei, and the green dots represent TLR2 (Magnification × 100). (**B**) Western blot. *n* = 3. ^##^ *p* < 0.01, ^###^ *p* < 0.001 versus control group; * *p* < 0.05, ** *p* < 0.01 versus LAMPs group.

The level of TLR2 protein in mice lung tissues was detected using immunofluorescence staining, which indicated that TLR2 expression in mice lung tissue in the control group and TFCO-treated group was low. However, it was significantly higher after intranasal infection with *M. pneumoniae* (Figure 4). TFCO treatment could reduce the *M. pneumoniae*-induced increase in TLR2 expression in a dose-dependent manner (Figure 4). TLR2 expression in RAW264.7 cells was measured by immunofluorescence staining and Western blot, which also showed that administration of TFCO could dose-dependently decrease the elevated expression of TLR2 protein in RAW264.7 macrophages caused by LAMPs stimulation (Figure 5).

### 2.5. TFCO Attenuated the Activation of NF-κB Pathway in LAMPs-Stimulated RAW264.7 Cells

**Figure 6:**
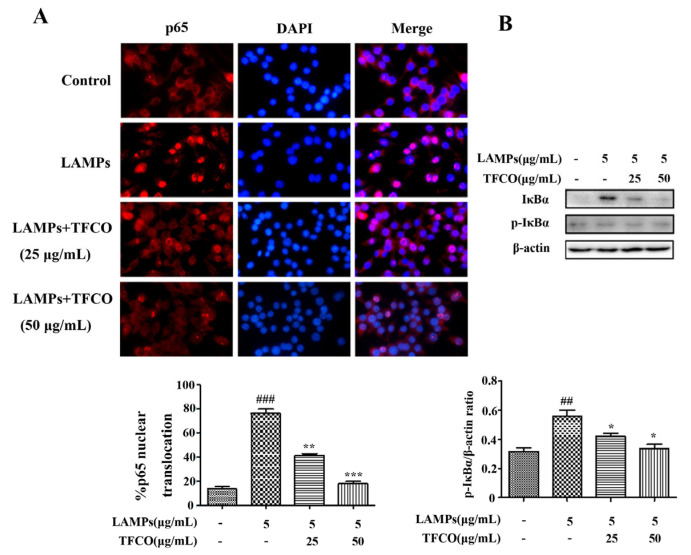
Effects of TFCO on the NF-κB signaling pathway in LAMPs-stimulated RAW264.7 cells (*n* = 3). (**A**) the translocation of p65 into nucleus: DAPI stained blue dots represent nuclei, and the red dots represent p65 (Magnification × 100). (**B**) Western blot: The phosphorylation of IκBα; ^##^ *p* < 0.01, ^###^ *p* < 0.001 versus control group; * *p* < 0.05, ** *p* < 0.01, *** *p* < 0.001 versus model group.

To further investigate the effects of TFCO on the *M. pneumoniae*-induced NF-κB signaling pathway, the contents of p-IκBα proteins in RAW264.7 macrophage cells were detected by Western blot assays. The results indicated that the p-IκBα proteins were markedly higher in the LAMPs-stimulated group compared to the control group. TFCO treatment dose-dependently reduced the expression of p-IκBα (Figure 6B). To verify our findings, we observed the nuclear translocation of the NF-κB p65 protein in RAW264.7 cells using immunofluorescence staining. The results indicated that after TFCO treatment, the nuclear p65 expression was significantly decreased compared to *M. pneumoniae*-challenged cells (Figure 6A).

### 2.6. TFCO Inhibited the Activation of MAPK Pathway in LAMPs-Stimulated RAW264.7 Cells

**Figure 7:**
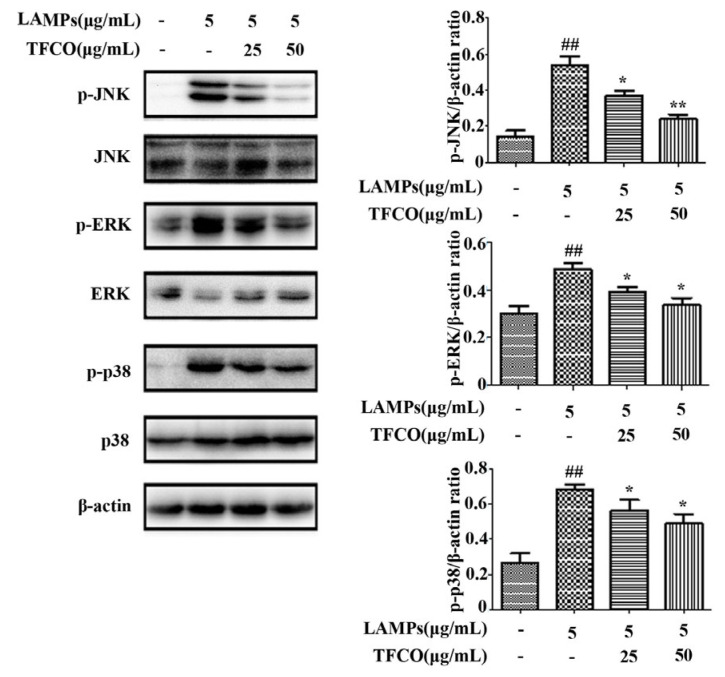
Effects of TFCO on the phosphorylation of MAPK signaling pathway in the LAMPs-stimulated RAW264.7 cells (*n* = 3). ^##^ *p* < 0.01 versus control group; * *p* < 0.05, ** *p* < 0.01 versus model group.

To assess the effect of TFCO treatment on the MAPK signaling pathway, the expression of proteins ERK, JNK, and p38 was determined. Our results revealed that expression levels of phosphorylated ERK, JNK, and p38 proteins were significantly increased in the LAMPs-stimulated group in comparison to the control group. In contrast, the expression of these phosphorylated proteins was significantly reduced by TFCO treatment in a dose-dependent pattern (Figure 7), indicating that TFCO might inhibit the MAPK signaling pathway to alleviate *M. pneumoniae* infection.

## 3. Discussion

*M. pneumoniae* is an obligate bacterial pathogen, particularly nefarious for its capacity to instigate respiratory diseases. When the epidemiological data are brought under the lens, it is evident that during outbreak seasons, *M. pneumoniae* shoulders the burden for 8% of CAP cases in pediatric populations and notable 2% in adults [32]. These statistics become even more compelling when viewed in a global context; *M. pneumoniae* outbreaks are not confined to specific geographies. Indeed, it has cast its shadow worldwide. Compounding this challenge is the rise of macrolide-resistant *M. pneumoniae* strains. Notably, regions such as Asia, Europe, and North America are sounding alarms with escalating prevalence rates of these resistant strains. Yet, the pathogenic repertoire of *M. pneumoniae* is not restricted merely to the respiratory tract. It is also implicated in a range of extrapulmonary manifestations, including damage to the skin, nervous system, heart, and joints [32].

*C. oleifera* is a highly valued plant with both medicinal and edible properties. The mature seeds of *C. oleifera* are mainly used for extraction of Camellia oil, which is a pure natural edible oil which has been used for treating gastrointestinal pain and skin burns in people for a long time [26]. The residues of *C. oleifera* seeds are by-products of oil production, traditionally used as animal feed or incinerated for heating. Pharmacological studies have shown that the chemical constituents from the residues of *C. oleifera* seeds possess a variety of biological activities, including anti-inflammation, antioxidation, antitumor, antibacterial, and so on [16,26]. Previous reports had reported that the hot-water extract of defatted *C. oleifera* seeds exerted significant anti-inflammatory effects, providing mild alleviation of hepatic inflammation in CCl_4_-induced liver hepatotoxicity [29]. Kaempferol glycosides, identified as the primary anti-inflammatory flavonoids in *C. oleifera* defatted seeds, have exhibited pronounced anti-inflammatory activity, corroborating our findings [20,21]. These glycosides displayed significant anti-inflammatory effects in mouse asthma [33,34] and rat transient focal stroke models [35]. In this study, we first investigated the protective effects of kaempferol glycosides against *M. pneumoniae*-induced lung injury in mice.

Immune damage resulting from the excessive secretion of inflammatory factors plays a crucial role in the pathogenesis of *M. pneumoniae* infection. A hallmark of *M. pneumoniae*’s biology is its absence of a traditional cell wall. This anomaly directs attention towards LAMPs anchored in its membrane, which are increasingly recognized as key pathogenic triggers. These proteins interface with TLR2 receptors on monocytes and macrophages, setting off a cascade of immune reactions [7]. This liaison stimulates the copious release of inflammatory mediators, forging a path towards immune-mediated tissue damage [7]. In this study, the in vivo model of *M. pneumoniae* pneumonia was established by infecting mice with *M. pneumonia* nasal drops [36,37], while the in vitro model was built by stimulating RAW264.7 macrophages with LAMPs.

Several studies have confirmed that the secretion of pro-inflammatory cytokines, such as TNF-α, IL-1β, and IL-6, is closely related to the severity and outcome of diseases following *M. pneumoniae* infection. Their biphasic role is evident: at physiological levels, they fine-tune immune responses aiding in pathogen elimination; however, when secreted in excess, they can wreak havoc, spurring uncontrolled inflammation, cellular death, and extensive tissue necrosis [38,39,40]. Previous studies highlighted the substantial inhibitory effects of kaempferol glycosides, isolated from *C. oleifera* defatted seeds and other plants, on the production of proinflammatory cytokines in LPS-activated macrophages [20,21,41,42]. In our investigation, the level of TNF-α, IL-1β, and IL-6 in the BALF from *M. pneumoniae*-infected mice, as well as in the supernatant from LAMPs-stimulated RAW264.7 macrophages, were notably reduced in the TFCO-treated cohorts compared to the model groups, exhibiting a dose-dependent trend. These findings suggested that the anti-inflammatory effects of TFCO, primarily kaempferol glycosides, may be related to the reduction in pro-inflammatory levels.

TLR is a transmembrane pattern recognition receptor that can be activated by invading pathogens, mediating immune response, and participating in inflammatory reactions. TLR2, as the most crucial pattern recognition receptor for immune system stimulation by *M. pneumoniae*, is the main receptor for LAMPs. However, it cannot recognize LAMPs alone and requires the assistance of other TLRs. Specifically, TLR2 collaborates with TLR1 to recognize triacyl lipoproteins and with TLR6 to recognize diacyl lipoproteins [14,43]. Studies have shown that TLR2 is closely related to lung inflammatory damage caused by *M. pneumoniae* [7,44]. In Balb/c mice infected with *M. pneumoniae*, the activation of TLR2 significantly upregulates the secretion of respiratory mucin [44]. Moreover, after mice have TLR2 knocked out or are intranasally inoculated with TLR2 antibodies, the secretion of inflammatory factors in their BALF is significantly reduced, further indicating that TLR2 activation is an essential factor inducing inflammatory reactions post *M. pneumoniae* infection [44]. In this experiment, following the infection of Balb/c mice with *M. pneumoniae* or stimulation of mouse macrophages RAW264.7 with LAMPs, a noticeable increase was observed in TLR2 protein expression in mouse lung tissues and RAW264.7 cells. After being treated with different concentrations of TFCO, the expression of TLR2 significantly decreased in a dose-dependent manner. Consequently, we posited that TFCO primarily alleviated pulmonary inflammation and damage caused by *M. pneumoniae* infection by inhibiting TLR2 expression.

To further investigate the key mechanisms underlying the anti-inflammatory effects of TFCO, we examined the TLR2-mediated activation of the NF-κB and MAPK signaling pathways. Both the NF-κB and MAPK pathways are highly conserved signaling pathways present in all eukaryotes, participating in a wide range of essential cellular physiological and pathological processes, such as regulating cell growth, differentiation, and inflammatory responses. Upon recognition by TLR2, LAMPs can activate NF-κB and MAPKs to induce the production of various pro-inflammatory cytokines and mediators in immune cells, such as monocytes, macrophages, and natural killer cells, participating in immune responses [45]. The MAPK signaling pathway mainly includes three pathways: JNK, ERK, and p38. Previous literature has shown that in RAW264.7 cells stimulated with *M. pneumoniae* extracts or infected with *M. pneumoniae*, all three signaling pathways are activated, which is consistent with our results [12,46]. TFCO treatment remarkably alleviated the phosphorylation of NF-κB and MAPK signaling pathway proteins both in vivo and in vitro.

While kaempferol glycosides constitute the primary component of TFCO at 98.44%, the potential influence of other active ingredients on the experimental outcomes should not be overlooked. The bioactive compounds in *C. oleifera* defatted seeds predominantly include flavonoids [17,18,19,20,21], saponins [22,23], and polysaccharides [24,25]. Notably, saponins obtained from *C. oleifera* possessed in vivo anti-inflammatory activity [47,48]. While no anti-inflammatory activity has been reported for polysaccharides from *C. oleifera*, the polysaccharides from other plants have shown significant anti-inflammatory properties [49]. Thus, the residual saponins and polysaccharides in TFCO might also contribute to its anti-inflammatory effect on *M. pneumoniae* pneumonia in mice.

Beyond *M. pneumoniae* pneumonia, the total flavonoids from *C. oleifera* defatted seeds might benefit in the treatment of other conditions, particularly those related to TLR2 activation, like sepsis and Clostridium difficile infection [50]. Additionally, kaempferol glycosides in TFCO might possess similar effects to their derivatives, which have mitigated inflammation in asthma and stroke in vivo [33,34].

## 4. Materials and Methods

### 4.1. Reagents and Antibodies

DMEM, PBS, and fetal bovine serum (FBS) were purchased from Gibco (Grand Island, NY, USA). Rabbit antibodies against TLR2, cy3-conjugated secondary antibody, 4′, 6-diamidino-2-phenylindole (DAPI) were purchased from Abcam (Cambridge, UK). Rabbit antibodies against p65, p-IκBα, IκBα, p-JNK, JNK, p-ERK, ERK, p-p38, p38, mouse antibody β-actin, and HRP-labeled secondary antibodies were products of Cell Signaling Technology (Beverly, MA, USA). The ELISA assays kit for TNF-α, IL-1β, and IL-6 were bought from Thermo Fisher (Invitrogen, Waltham, MA, USA). The pleuropneumonialike organisms (PPLO) broth was obtained from BD Biosciences (Franklin Lake, NJ, USA). Triton X-114 was product of Solarbio (Beijing, China). HRESIMS were measured using AB SCIEX Triple TOF 6600 mass instruments (SCIEX, Framingham, MA, USA). UPLC was performed on Agilent 1290 (Agilent, Palo Alto, CA, USA). Silica gel (200–300 mesh, Qingdao, China), HP-20 macroporous resin (Lupital, Japan) were utilized for column chromatography. Subsequently, ODS column chromatography was applied to obtain compound **1** and compound **3** in TFCO.

### 4.2. Preparation and UPLC-HRESIMS Analysis of TFCO

Five kilograms of *C. oleifera* seeds, collected from factories in Youxian County, Hunan Province, China, were ground into a powder and defatted using petroleum ether and ethyl acetate [18,21]. Subsequently, TFCO was prepared from the butyl alcohol extract of *C. oleifera* seeds, which was treated with a silica gel (200–300 mesh) column chromatography elution with a gradient CH_2_Cl_2_-MeOH solvent system (from 10:1 to 1:1, and finally only MeOH), followed by HP-20 macroporous resin eluted with 70% ethanol.

Chromatographic separation of TFCO was conducted on an Acquity HSS T3 C18 column (2.1 mm × 100 mm, 1.8 μm, Waters). The gradient elution system comprised solvent A (formic acid/water, 0.5:99.5, *v*/*v*) and solvent B (acetonitrile) and was set as follows: 0−10 min, 5−25% B; 10−13 min, 25−50% B; 13−14 min, 50−95% B. The flow rate was 0.3 mL/min. The ions of HRESIMS were obtained within the range of *m/z* 100–1500.

### 4.3. M. pneumoniae Culture and Extraction of LAMPs

The *M. pneumoniae* culture and extraction of LAMPs were carried out following previously described methods [28]. Briefly, *M. pneumoniae* strain M129 (ATCC 29342) was cultured in PPLO broth (BD Biosciences) supplemented with 20% FBS, 0.25% glucose, 0.4% yeast extract (Oxoid), penicillin G (1000 U/mL), and phenol red (0.005%) at pH 7.6 and 37 °C for 5–7 days, until the pH indicator transitioned from red to orange. The *M. pneumoniae* pellet was obtained by centrifugation, resuspended in Tris-buffered saline with 1 mM ethylenediaminetetraacetic, and further dissolved by Triton X-114 (Solarbio, Beijing, China) to a final concentration of 2%. Subsequently, the Triton X-114 lysate was incubated at 37 °C for 10 min to allow phase separation and the underlying Triton X-114 phase was retained. Precipitated protein was obtained by further adding ethanol and centrifugation. Finally, the LAMPs were resuspended in phosphate-buffered saline (PBS) to determine concentration and stored at −20 °C or −80 °C for subsequent experiments.

### 4.4. Animals and Experimental Design

Specific-pathogen-free Balb/c mice (male, 4 week-old) were acquired from SJA Laboratory Animal Co., Ltd. (Changsha, China) and assigned into five groups (*n* = 5/group): the control group, the TFCO group (mice treated with 100 mg/kg TFCO only), the *M. pneumoniae* group (*M. pneumoniae*-infected mice), the *M. pneumoniae* + low TFCO group (*M. pneumoniae*-infected mice treated with 50 mg/kg TFCO), and the *M. pneumoniae* + high TFCO group (*M. pneumoniae*-infected mice treated with 100 mg/kg TFCO). Mice in the control and TFCO groups were treated with PPLO using nasal drops, whereas mice in the other groups were intranasally inoculated with 50 μL *M. pneumoniae* suspension (1 × 10^8^ CFU/mL) for 3 days. Additionally, TFCO was administered by gavage in the TFCO, *M. pneumoniae* + low TFCO, and *M. pneumoniae* + high TFCO groups once at 10 am daily for 7 consecutive days. The control and *M. pneumoniae* groups received intragastric administration of 200 µL of PBS.

On day 7, mice were euthanized and fixed in a supine position. The lungs were irrigated three times with 0.5 mL of PBS to obtain the BALF for pro-inflammatory cytokines determination. The entire lungs were collected for HE staining and immunohistochemistry. All animal experiments were approved by the University of South China Institutional Animal Use and Ethics Committee (Hengyang, China).

### 4.5. Cell Culture and Stimulation with LAMPs

The mouse macrophage cell line RAW264.7 was purchased from the Cell Bank of the Chinese Academy of Sciences (Shanghai, China) and cultivated in DMEM (HyClone, Logan, UT, USA) containing 10% FBS, 2 mmol/L L-glutamine, and 100 μg/mL penicillin/streptomycin. Cells were seeded in 6-well plates at a density of 1 × 10^6^ cells/well. After incubating at 37 °C under 5% CO_2_ for 24 h, the TFCO (50 μg/mL, 25 μg/mL) was added to the wells and cultured for 1 h. Then, cells were stimulated with or without 5 μg/mL LAMPs for 24 h.

### 4.6. Cell Viability Assay

The cytotoxicity of TFCO was assessed using CCK-8 assay. The cells (2 × 10^5^ cells/well) were seeded in 96-well plates and cultured overnight. The cells were incubated for 2 h with or without TFCO (12.5, 25, 50 and 100 µg/mL) and in the presence or absence of LAMPs (5 µg/mL). Following a 24 h incubation, 10 µL of CCK-8 solution was added to each well, and the absorbance optical density (OD) was measured at a wavelength of 450 nm using an enzyme labeling instrument.

### 4.7. HE Staining

The left lung lobes were fixed with 4% paraformaldehyde, embedded in paraffin, and sectioned. The sections were stained by HE for observation under light microscopy. Lung injury severity was assessed using a semiquantitative histology score through a double-blind method. The histopathological score (HPS) was determined based on the condition of peribronchial or bronchial infiltration, bronchial or bronchial exudate, perivascular and leukocyte infiltration. Each item was assigned a grade ranging from 0 to 3 (0 = no damage; 1 = mild damage; 2 = moderate damage; and 3 = severe damage). The calculated scores were used for statistical analysis.

### 4.8. Enzyme-Linked Immunosorbent Assay

TNF-α, IL-6, and IL-1β levels in mouse BALF and the supernatants of RAW264.7 cells were measured using commercially available enzyme immunoassay kits (Invitrogen, Waltham, MA, USA) according to the manufacturer’s instructions.

### 4.9. Immunofluorescence Staining

Lung tissue sections were deparaffinized in xylene and dehydrated with graded ethanol. Heat-mediated antigen retrieval was conducted in EDTA antigen repair buffer (pH 6.0), followed by permeabilization for 10 min with 0.1% Triton X-100, and blockage for 1 h in 5% bovine serum albumin. Subsequently, lung sections were incubated with rabbit antibody against TLR2 at 4 °C overnight, and then stained with Cy3-conjugated goat-anti-rabbit antibody at room temperature for 1 h. After washing in PBS and staining with DAPI, lung sections were examined and images were captured using a fluorescence microscope (Nikon Eclipse C1, Tokyo, Japan).

Mouse RAW264.7 cells were seeded onto coverslips in a 24-well plate. After fixing with 4% PFA for 30 min and permeabilizing with 0.3% Triton X-100 for 10 min, cells were blocked with DMEM containing 10% FBS. Subsequently, the specimens were incubated with primary rabbit antibody against TLR2 and NF-κB p65 overnight at 4 °C, followed by incubation with Cy3-conjugated goat anti-rabbit antibody for another 1 h. Nuclei were stained with DAPI for 15 min at room temperature and images were acquired using an immunofluorescence microscope (Ts2R, Nikon, Japan).

### 4.10. Western Blot Analysis

RAW264.7 cells for Western blot assay were collected and lysed by RIPA lysis buffer containing 1% phosphatase and protease inhibitors. Protein content in the samples was measured by BCA protein assay kit. After denaturing in boiling water for 10 min, equal amounts of the total proteins were separated by SDS-PAGE electrophoresis and electro-transferred to PVDF membranes following standard procedures. The membranes were blocked with TBST containing 5% non-fat milk for 2 h at room temperature and incubated with primary antibodies against β-actin, TLR2, p-IκBα, IκBα, p-JNK, JNK, p-ERK, ERK, p-p38, and p38 overnight at 4 °C. After washing with TBST five times, HRP-conjugated secondary antibody was added to the membranes for 1 h at 37 °C, followed by further washing with PBS. Immunoreactive bands were visualized using the ECL system (Syngene, G: BOX XX8, Cambridge, UK) and densitometric analysis was quantified using Image J 1.44.

### 4.11. Statistical Analysis

All experiments were performed independently three times, and the data referenced above were expressed as Mean ± SEM. GraphPad Prism 5.0 software was used for statistical analysis, and one-way ANOVA was applied to compare different groups. Statistical significance was defined as * *p* < 0.05, ** *p* < 0.01, and *** *p* < 0.01.

## 5. Conclusions

In summary, our study is the first to investigate the protective effects of kaempferol glycosides, including, but not limited to, those from *C. oleifera* defatted seeds, against *M. pneumoniae*-induced lung damage. The potential mechanism may be associated with the inhibition of TLR2-medicated NF-κB and MAPK signaling pathways. This research underscored the potential therapeutic value of *C. oleifera*, suggesting its prospective use in treating lung inflammation caused by *M. pneumoniae.*

## Data Availability

The data presented in this study are available from the corresponding author upon reasonable request.

## Supplementary Materials

The following supporting information can be downloaded at: https://www.mdpi.com/article/10.3390/molecules28207077/s1. Table S1. ^1^H NMR (600 MHz) and ^13^C NMR (150 MHz) data for compounds **1** and **3** in CD_3_OD (*δ* in ppm, *J* in Hz). Table S2. Contents of TFCO and compounds **1–5** in *Camellia oleifera* seed. Figure S1. The load of *M. pneumoniae* in the lungs of infected mice. ns: non-significant differences versus *M. pneumoniae* group. Figure S2. The weight of mice. ns: non-significant differences, ^**^*p* < 0.01. Figure S3. ^1^H NMR of compound **1**. Figure S4. ^13^C NMR of compound **1**. Figure S5. ^1^H NMR of compound **3**. Figure S6. ^13^C NMR of compound **3**.

## Sample Availability

Samples of TFCO are available from the authors.
