# Supplementary material for: Total Flavonoids from Camellia oleifera Alleviated Mycoplasma pneumoniae-Induced Lung Injury via Inhibition of the TLR2-Mediated NF-κB and MAPK Pathways
Source: Molecules. 2023 Oct 13;28(20):7077. doi: 10.3390/molecules28207077 (PMC10609408; doi:10.3390/molecules28207077)
Supplement: Supplementary file 1 [file molecules-28-07077-s001.zip › molecules-2639014-supplementary.pdf]

Table S1. ^{1}H NMR (600 MHz) and ^{13}C NMR (150 MHz) data for compounds **1** and **3** in CD_3OD (δ in ppm, J in Hz)

No.	**1**		**3**	
	δ_C	δ_H	δ_C	δ_H
2	157.2		157.2	
3	133.3		133.4	
4	178.1		177.9	
5	161.6		161.6	
6	98.7	6.18 d (1.8)	98.9	6.16 s
7	165.1		165.7	
8	93.7	6.38 d (1.4)	93.7	6.34 s
9	160.1		160.0	
10	104.2		104.0	
1’	121.5		121.6	
2’, 6’	131.0	8.02 d (8.8)	131.0	8.05 d (8.8)
3’, 5’	114.8	6.90 d (8.8)	114.8	6.89 d (8.8)
4’	157.9		157.2	
	β-D-Gluco (A)		β-D-Gluco	
1	99.8	5.34 d (7.6)	99.5	5.33 d (7.5)
2	80.6		80.5	
3	76.9		76.8	
4	70.7		70.7	
5	76.4		75.6	
6	66.9		66.7	
	α-L-Rhamno		α-L-Rhamno	
1	100.8	4.48 d (1.4)	100.8	4.49 s
2	70.9		70.9	
3	72.5		72.5	
4	70.0		70.0	
5	68.3		68.3	
6	16.5	1.09 d (6.2)	16.5	1.09 d (6.2)
	β-D-Gluco (B)		β-D-Xylo	
1	103.1	4.77 d (7.4)	103.8	4.78 d (6.8)
2	74.0		73.3	
3	75.5		75.5	
4	69.9		69.6	
5	76.5		65.2	
6	61.2			

Table S2. Contents of TFCO and compounds **1-5** in *Camellia oleifera* seed.

	Total Yield	% Yield (*w*/*w*)
TFCO	28.40	0.57
Compound **1**	11.20	0.22
Compound **2**	1.07	0.02
Compound **3**	15.04	0.30
Compound **4**	0.37	＜0.01
Compound **5**	0.27	＜0.01

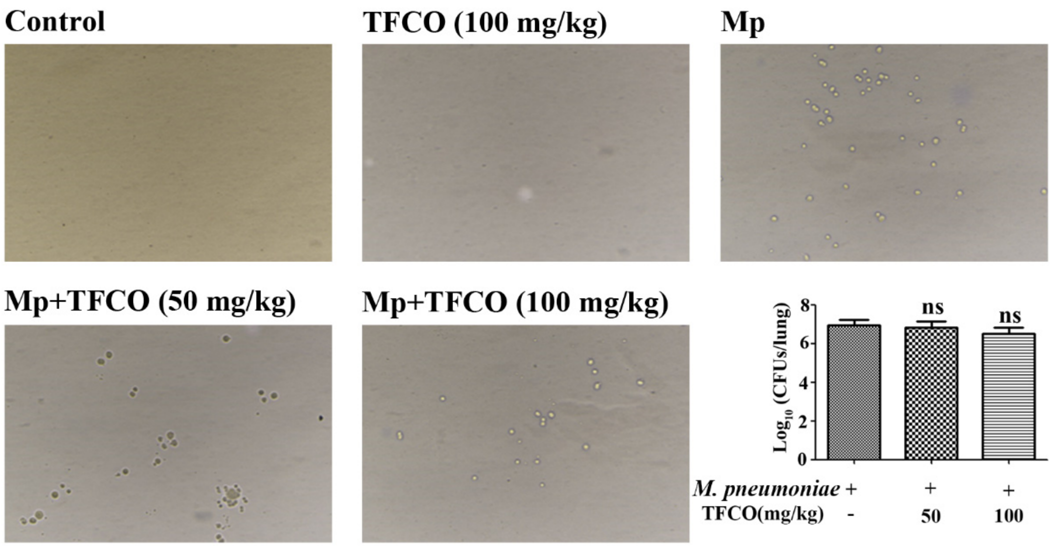

Figure S1. The load of *M. pneumoniae* in the lungs of infected mice. ns: non-significant differences versus *M. pneumoniae* group

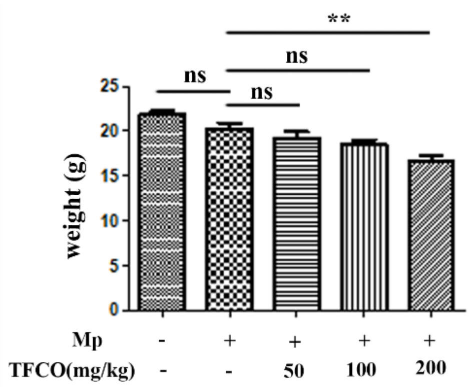

Figure S2. The weight of mice. ns: non-significant differences, $^{**}p < 0.01$.

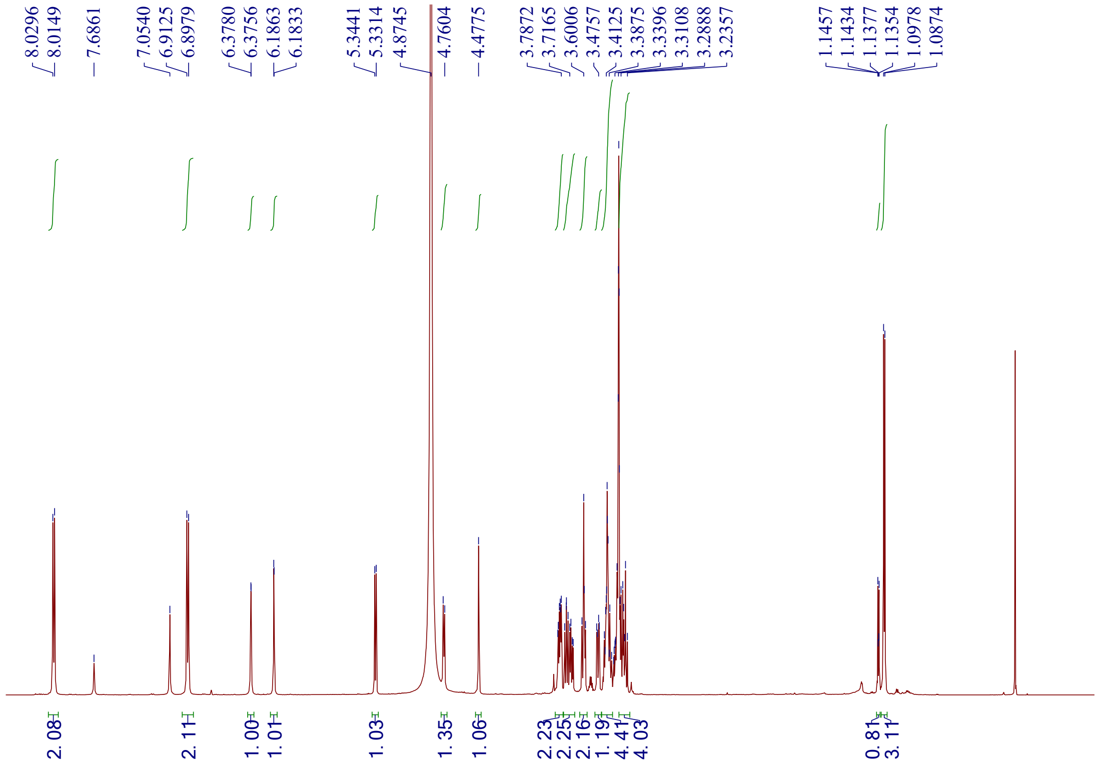

Figure S3. ^{1}H NMR of compound **1**

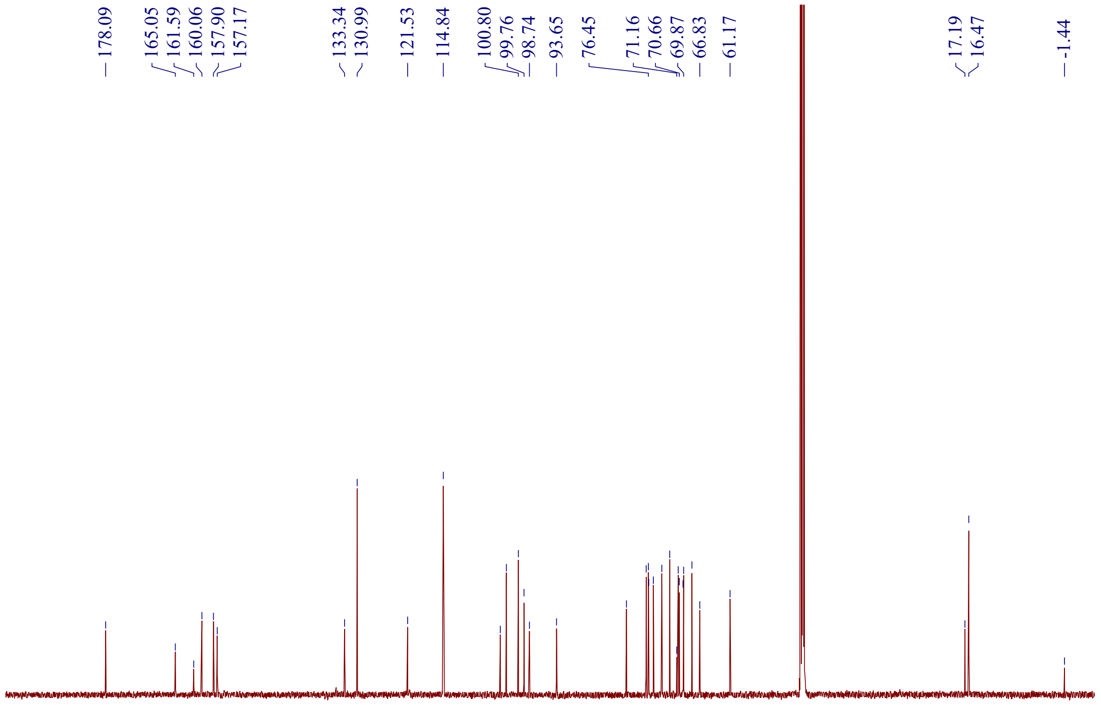

Figure S4. ^{13}C NMR of compound **1**

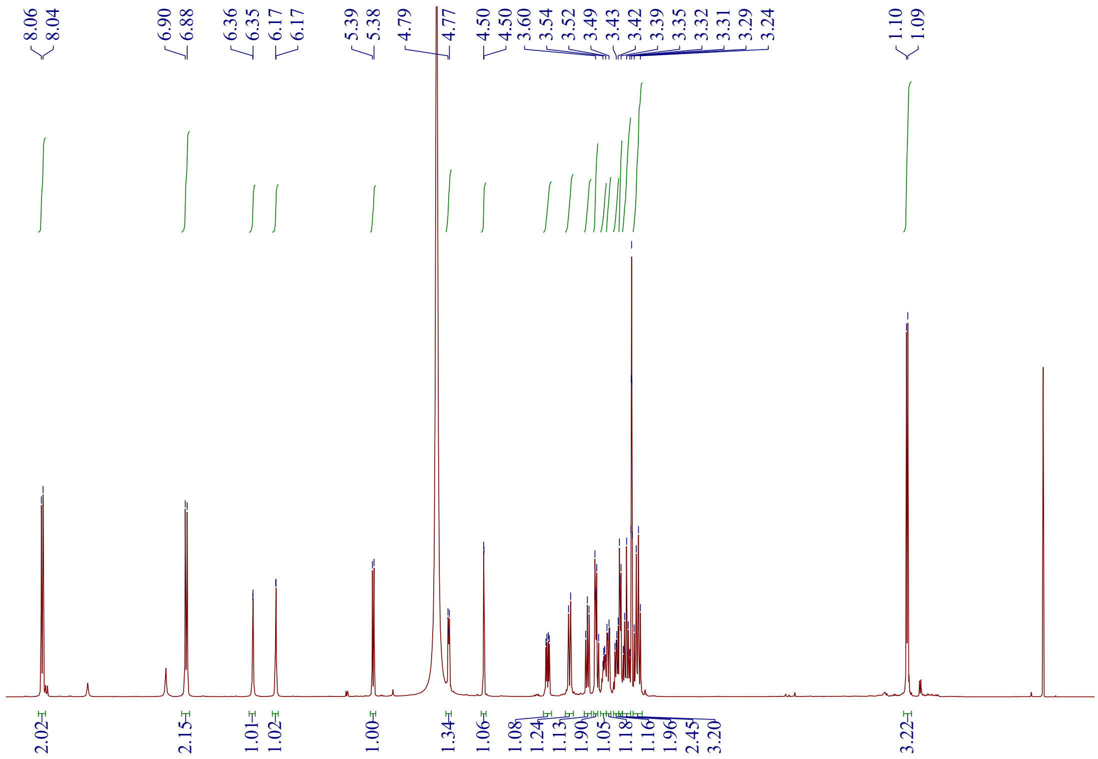

Figure S5. ^{1}H NMR of compound **3**

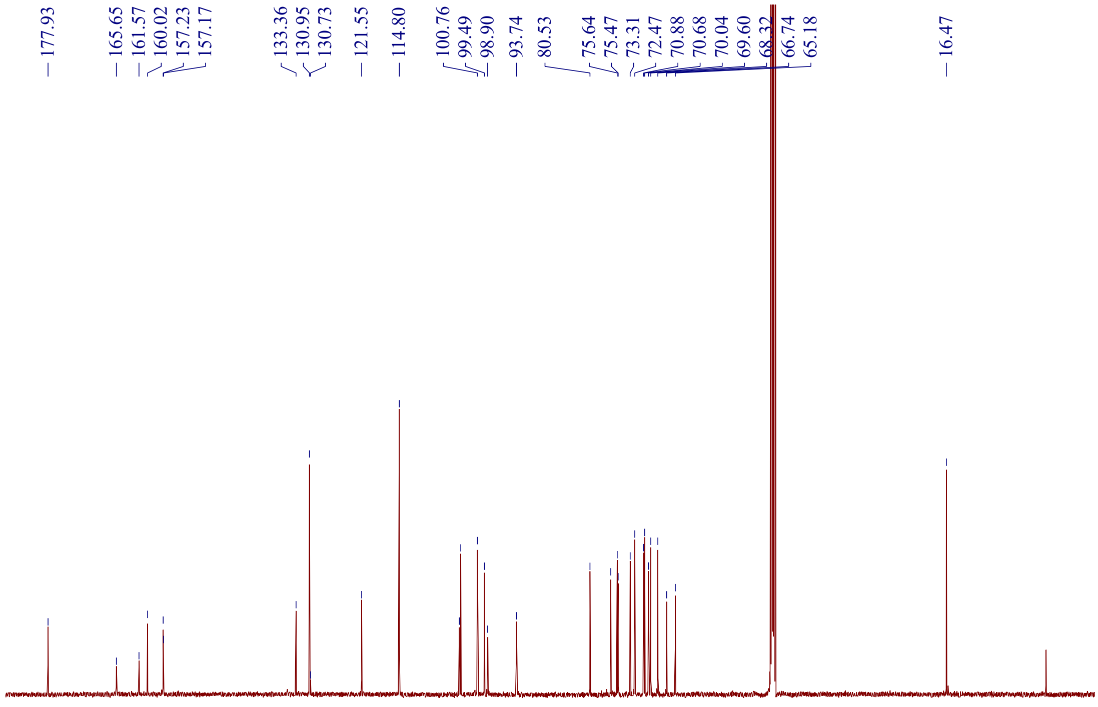

Figure S6. ^{13}C NMR of compound **3**
